# Pelvic Radiation Disease Management by Hyperbaric Oxygen Therapy: Prospective Study of 44 Patients

**DOI:** 10.1155/2014/108073

**Published:** 2014-01-27

**Authors:** Mehdi Ouaïssi, Stephanie Tran, Diane Mege, Vivien Latrasse, Alain Barthelemy, Nicolas Pirro, Philippe Grandval, James Lassey, Igor Sielezneff, Bernard Sastre, Mathieu Coulange

**Affiliations:** ^1^Aix-Marseille University, UMR 911, Campus Santé Timone, 13005 Marseille, France; ^2^Department of Digestive Surgery, AP-HM Timone Hospital, Pôle DACCORD, 13385 Marseille, France; ^3^Atelier Provençal d'écriture Médicale, France; ^4^Hyperbaric Medicine, Sainte Marguerite Hospital, Aix-Marseille University, UMR MD2, 13385 Marseille, France; ^5^Department of Gastroenterology, AP-HM Timone Hospital, Pôle DACCORD, 13385 Marseille, France

## Abstract

Pelvic radiation disease (PRD) occurs in 2–11% of patients undergoing pelvic radiation for urologic and gynecologic malignancies. Hyperbaric oxygen therapy (HBOT) has previously been described as a noninvasive therapeutic option for the treatment of PRD. the purpose of study was to analyze prospectively the results of HBOT in 44 consecutive patients with PRD who were resistant to conventional oral or topical treatments. *Material and Methods*. The median age of the cohort was 65.7 years (39–85). Twenty-seven percent of patients required blood transfusion (*n* = 12). The median of delay between radiotherapy and HBOT was 26 months (3–175). We evaluated the results of HBOT, using SOMA-LENT Scale. *Results*. SOMA-LENT score was decreased in 59% of patient. The median of SOMA-LENT score before HBOT was significantly higher, being equal to 14 (0–36), than after HBOT with the SOMA-LENT score of 12 (0–38) (*P* = 0.003). Tenesmus (*P* = 0.02), bleeding (*P* = 0.0001), and ulceration (*P* = 0.001) significantly decreased after HBOT. Regarding patients with colostomy, 33% (*n* = 4) benefited from colostomies closure. HBOT was generally well tolerated. Only one patient stopped precociously due to transient myopia. *Conclusion*. This study is in favor of the interest of HBOT in pelvic radiation disease treatment (PRD).

## 1. Introduction

Pelvic radiation disease (PRD) induced by radiation occurs in 2–11% of patients undergoing pelvic radiation for urologic and gynecologic malignancies [1–4]. PRD occurs 3 months after radiotherapy and is characterized by the painless passage of blood per rectum (clots or streaking of the stool), mucous rectal discharge, frequent bowel movements, and rectal pain. Less commonly, bowel obstruction, fistulae, bowel perforation, and severe rectal bleeding requiring blood transfusions are observed. Treatments for PRD are not universally successful. Current modalities include pharmacological agents such as oral and rectal steroids, 5-amino salicylates, sucralfate, short chain fatty acid enemas, oral metronidazole, and oral vitamins E and C [5, 6]. Acute hemorrhage could imply local haemostatic treatments including topical formalin, yttrium-aluminum-garnet (YAG), laser and/or surgical intervention consisting of defunctioning colostomy in severe cases [5]. Hyperbaric oxygen therapy (HBOT) has previously been described as a noninvasive therapeutic option for the treatment of radiation PRD [7]. HBOT is thought to promote neovascularization by improving oxygenation thus increasing the p02 and promoting wound healing in the damaged rectal mucosa, thereby reducing bleeding [8]. Cochrane's review concludes that HBOT in late tissue radiation injury is associated with improved outcome though the review was based on small trial series. In the present study, we are assessing the efficacy of HBOT in 44 consecutive patients with PRD who were resistant to conventional oral or topical treatments [6].

## 2. Materials and Methods

### 2.1. Type of Study

Between January 2001 and December 2009, forty-four patients with PRD, from the department of digestive surgery and hyperbaric Medicine unit prospectively included in this study and treated with HBOT. Before treatment, PRD was confirmed by sigmoidoscopy and biopsy in all patients.

### 2.2. Types of Participants

We included all patients with PRD following radiation therapy for pelvic cancer. The patient characteristics are listed in Table 1. There were anal cancer (*n* = 18%), prostate cancer (*n* = 41%), uterine cancer (*n* = 29%), and other types of cancer (*n* = 11%) (Table 1). Diagnosis of PRD was established by endoscopy showing edematous and inflamed mucosa. PRD symptoms are tenesmus, rectal bleeding, stenosis, occlusion/constipation, fistulas, and incontinence. All patients were free of oncological disease.

### 2.3. Types of Outcome Measures

The Undersea and Hyperbaric Medical Society defines HBOT as a treatment where a patient intermittently breathes 100% oxygen while the treatment chamber is pressurized to greater than sea level (1 absolute atm ATA). HBOT is thought to promote neovascularization by improving oxygenation thus increasing the pO2 to the damaged tissue, normalizing the tissue, and promoting wound healing to the damaged rectal mucosae, thereby reducing bleeding.

All patients received HBOT as a treatment of their PRD. The chamber was pressurized to 2.5 absolute atm and patients were treated for 60 minutes with 100% oxygen. Before HBOT, a history and physical investigation was performed by HBOT-trained specialists to validate the indication and eliminate the contraindications to HBO. Standard administration of HBOT was 20 sessions and was continued until a decrease in symptoms was observed. Treatment was given on a once-a-day basis, 5 to 7 days a week.

The pretreatment characteristics of the patients, the type of radiation received, SOMA-LENT score [9], the number of HBOT, the timing of the onset and reduction of symptoms, endoscopic injuries, the severity of the CRP, and the toxicity of treatment were collected prospectively.

The SOMA-LENT Scale (Table 2) [9] evaluates three different fields. First, subjective symptoms including tenesmus, mucus loss, defecation frequency, and pain and then objective symptoms including bleeding, ulceration, and stenosis are assessed. The third part details the medical management of all the symptoms (tenesmus and defecation frequency, pain, bleeding, ulceration, stenosis, and incontinence). The SOMA-LENT Scale defines 4 grades of increasing severity, of each criterion. The score can range from 0 to 56.

### 2.4. Statistical Analysis

Values are expressed as mean, median, and range. Statistical analysis was performed using GraphPad Software. Data are expressed as mean ± standard deviation or median with interquartile range. The differences between the two groups were analyzed using the Mann-Whitney *U* test or Student's *t*-test. One-way analysis of variance or Kruskal-Wallis test was performed to compare more than two groups. Multivariate survival analysis using Cox's regression model was conducted. To compare categorical variables, the chi-square or Fisher exact test was used. Kaplan-Meier method was used to estimate overall and relapse-free survival. For all tests, a *P* value of less than 0.05 was considered significant.

## 3. Results

The median age of the cohort was 65.7 years (39–85). There were 18 females and 26 males. Median of delay between radiotherapy and the beginning of PRD symptoms was 16 months and the median of delay between radiotherapy and HBOT was 26 months (3–175). The median of followup was 8 months (3–17). PRD was associated chronic phase of radiation-induced damage in bladder for 17 patients (38%), in ileum for 2 patients (4%), and in sigmoid for 4 patients (9%).

Before HBOT, all patients had oral or topical treatments. Twenty-seven percent of patients required blood transfusion (*n* = 12). Electrocautery or argon plasma was performed on 13.6% (*n* = 6) of patients. Corticosteroids enema was used on 29.6% (*n* = 13) of patients; 6 of these patients also received formol enema. Twenty-seven percent (*n* = 12) of patients required colostomy due to anal incontinence (*n* = 4), stenosis (*n* = 4), and fistula or sepsis (*n* = 4). No medical treatment was performed during HBOT.

The median number of sessions was 35, ranging from 6 to 90. Only one patient stopped precociously the HBOT (after 6 sessions) due to transient myopia.

**Figure 1:**
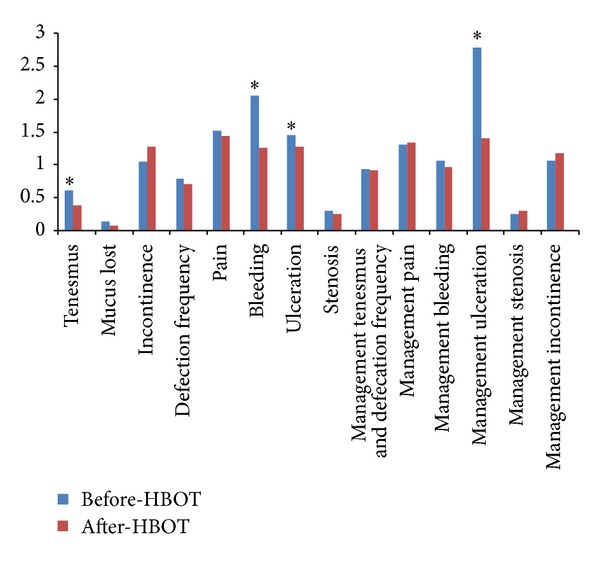
Median score for SOMA-LENT before HBOT versus SOMA-LENT after HBOT per patient and per symptom. *P < 0.05. HBOT: hyperbaric oxygen therapy. Managing bleeding: patient required instrumental and/or blood transfusion Bleeding: patients with acute rectorrhagia.

At the end of followup twenty-six patients (59%) had a decreased SOMA-LENT score after HBOT, ten patients (22.8%) had a SOMA-LENT score unmodified, and eight patients (18.3%) showed an increase of the SOMA-LENT score. The median of SOMA-LENT score before HBOT was significantly greater, 14 (0–36), than after HBOT with the SOMA-LENT score of 12 (0–38) (*P* = 0.003) (Figure 1).

HBOT decreased many symptoms: tenesmus, mucus loss, defecation frequency, bleeding, ulceration, and stenosis, with significant difference in tenesmus (*P* = 0.02), bleeding (*P* = 0.0001), ulceration (*P* = 0.001), and management of ulceration (*P* = 0.001) after HBOT (Figure 1). Concerning patients with colostomy, 33% (*n* = 4) benefited from colostomies closure.

HBOT was generally well tolerated. Four patients had transient hearing problems. No cancer recurrence was found.

## 4. Discussion

PRD is seen after radiotherapy for any pelvic malignancy, including that of the bladder, the prostate, and the uterus. Although the complex pathological process of radiation-induced injury to the rectum begins immediately following exposure, it may require weeks to months to become clinically apparent [3, 4]. Late effects of radiation are seen from damaged to slowly replicating cells and by the induction of proinflammatory and procoagulation cytokine signaling pathways, leading to edema, fibrosis, and ultimately ischemia in the muscularis [10]. It is estimated that up to 30% of patients undergoing pelvic radiotherapy have acute rectal toxicities with 15% of patients experiencing chronic symptoms [11].

There are no standard therapies for radiation-induced proctopathy and a number of treatments have been described with varying efficacy, including pharmacotherapy, sclerotherapy, and HBOT [5].

By increasing systemic oxygen partial pressure, HBOT increases the delivery of oxygen to ischemic tissues [12], thereby promoting angiogenesis, nutrient influx, and fibroblast proliferation [12]. Several small retrospective series suggest that hyperbaric oxygen can successfully treat radiation-induced proctopathy with response rates between 40% and 60% [13]. A recent randomized, placebo controlled trial showed statistically significant improvement in wound healing with hyperbaric oxygen in patients with late radiation tissue necrosis compared to patients receiving normal air at 2 atmospheres [14]. In our study we demonstrate that HBOT had a significant impact on decreasing tenesmus and hemorrhage. Many patients had a failed medical treatment before HBOT as reported in a randomized control trial [14].

Our study population consisted of patients with severe radiation proctopathy after radiotherapy for pelvic cancer in whom multiple attempts at management including steroid injection, anti-inflammatory suppositories, and argon plasma laser coagulation failed. Due to our relatively small sample size, no inferences can be made on the outcomes in relation to prior treatments received. However, rectal bleeding was significantly reduced with an improvement in more than half of the patients. Rectal ulcers showed favorable responses to HBOT with partial or complete resolution in 20% of patients.

We used a standard scoring system to directly compare our outcomes to those from other series. Fewer than 20% of patients with this degree of injury heal spontaneously. Our series showed a favorable response (60% improved) with HBOT in patients with severe radiation proctitis refractory to treatment. Improvements were seen in mucus lost, defecation frequency, pain, stenosis, bleeding, and ulceration, and especially in tenesmus, bleeding, and ulceration which seemed to be most improved.

The optimal dose of oxygen is not known. In our study, we used an average of 35 treatments. Using an average of 24 treatments (two atmospheres for 105 min per session), Woo et al. reported improvement in 10 of 18 study patients with radiation proctitis [15]. Furthermore, Feldmeier et al. have reported their experience in using HBOT in eight patients with large or small bowel injuries who were given a median number of 20 treatments, whereby 75% of those who had at least 22 treatments had complete resolution of their symptoms [16]. Shorter durations (60 min) to enhance patient compliance and 26 treatment sessions have been used by Mayer and colleagues [17]. This prospective study, although nonrandomized with a small sample size, suggests a significant and prolonged response with HBOT in a challenging patient population. Overall the prognosis is favorable and further studies will require long-term followup to determine the durability of response to HBOT [18].

The most common complications experienced with HBOT delivery are mild and transient, including barotraumatic otitis, confinement anxiety, temporary myopia, and euphoria. More severe effects include rare seizures from central nervous system oxygen toxicity and pulmonary oxygen toxicity. In a series of 782 patients undergoing HBOT, 17% experienced ear pain with 3.4% having visually confirmed barotraumatic otitis [19]. In our series of 44 patients, HBOT was extremely well tolerated with only one patient who stopped HBOT for reversible myopia. Otherwise, our study as in the literature [16] is not in favor of increased risk of cancer recurrence.

This study confirms the interest of HBOT in chronic radiation proctitis resistant to conventional treatments [20, 21]. A multicenter study comparing HBOT with conventional treatments would clarify the role of this indication.

## Figures and Tables

**Table 1:** Demographical data of population.

	*N* = 44 (%)
Age (years)	
Median	65.7 (39–85)
Gender	
Female	18 (41)
Male	26 (59)
Comorbidities	
Diabetes	2 (4.5)
Hypertension	16 (36.4)
Arteriopathy	9 (20.4)
Tabaco	16 (36.4)
Coronary disease	7 (15.9)
Corticotherapy	5 (11.3)
Immunosuppressive treatment	15 (34)

Type of cancer treated by radiation	
Prostate	18 (40.9)
Anal	8 (18.2)
Gynecologic cancer	
Uterus	8 (18.2)
Endometrial	5 (11.3)
Another	5 (11.3)

**Table 2:** SOMA-LENT Scale [9–22].

	Grade 1	Grade 2	Grade 3	Grade 4	Score
Subjective					
Tenesmus	Occasional urgency	Frequent urgency	Constant urgency	Nonresponder	
Mucus lost	Occasional	Frequent	Constant	Nonresponder	
Incontinence	Occasional	Frequent	Constant	Nonresponder	
Defecation frequency	2–4 times/day	4–8 times/day	>8 times/day	Incontrol diarrhea	
Pain	Occasional and minimal	Frequent and tolerable	Constant and intense	Nonresponder and atrocious	
Objective					
Bleeding	Hide	Occasional >2/week	Constant/daily	Brutal bleeding	
Ulceration	Superficial ≤1 cm²	Superficial >1 cm²	Deep ulcer	Perforation. Fistula	
Stenosis	>2/3 normal diameter without dilation	1/3 à 2/3 normal diameter with dilation	<1/3 normal diameter	Complete stenosis	
Management					
Tenesmus and defecation frequency	Occasional ≤2 antidiarrhea medication/week	Usually >2 antidiarrhea medication/week	Several >2 antidiarrhea medication/day	Surgery/colostomy	
Pain	Occasional, nonopiate treatment	Usually nonopiate treatment	Usually with opiate treatment	Surgery	
Bleeding	Laxatives, for treatment	Occasional blood transfusion	Frequent blood transfusion	Surgery/colostomy	
Ulceration	Dietary management, laxatives	Occasional corticosteroids	Steroids rectal injection, HBOT	Surgery/colostomy	
Stenosis	Dietary management	Occasional dilation	Usually dilation	Surgery	
Incontinence	Occasional use of protective pads	Frequent use of protective pads	Constant use of protective pads	Surgery/colostomy	
